# Cross-border prevalence of putative-hypervirulent *Klebsiella pneumoniae* differs between tertiary hospitals in Austria and Germany

**DOI:** 10.1016/j.nmni.2025.101606

**Published:** 2025-06-14

**Authors:** Bernd Neumann, Sophie Wilhelm, Jan Marco Kern, Joerg Steinmann

**Affiliations:** a Institute of Clinical Microbiology, Infectious Diseases and Infection Control, Paracelsus Medical University, Nuremberg General Hospital, Nuremberg, Germany; b Division of Medical Microbiology, Department of Laboratory Medicine, Paracelsus Medical University, Salzburg, Austria

**Keywords:** Hypervirulence, Cross-border surveillance, String-test, Diagnostics

## Abstract

We conducted a prospective testing protocol for surveillance of putative hypervirulent *Klebsiella pneumoniae* in two tertiary hospitals in Germany and Austria, over a one year study period. In total, 45 (1.02 %) putative hvKp of around 4400 *K. pneumoniae* were identified. The study revealed a 3.3-times higher prevalence of putative hvKp in the German hospital.

## Study and Observations

Hypervirulent *K. pneumoniae* (hvKp) can cause severe infections, such as liver abscesses, even in young and healthy patients. Identification of hvKp in microbiological diagnostics is difficult, especially due to varying definitions of hypervirulence in literature. Recently, Han et al. presented global data on hvKp prevalence and suggestions for definition for hvKp [1]. They highlight the obstacles of defining hvKp and thereby the difficulties for diagnostics and epidemiological investigations. Most hvKp isolates are susceptible to a broad range of antibiotics, so hypervirulence traits remain undetected unless a patient presents with a liver abscess. Due to this challenge, and the lack of general surveillance systems, the availability of data for Europe is sparse or solely based on single case reports.

To overcome the lack of structured epidemiological data in Central Europe, our study aimed to investigate the amount of putative hvKp in two tertiary hospitals. Thus, enabling the establishment of a surveillance network in two neighbouring countries and a cross-border region. For this purpose, a testing procedure in diagnostics was established over 12 months to pre-select hvKp. Both sites followed the general testing strategy as described previously [2]: in 2023, all clinical isolates of *K. pneumoniae* sent routinely to microbiology diagnostics department, regardless of patients’ case presentation or the kind specimen, were string-tested. String-test positive isolates were subjected for molecular investigation of hvKp-related virulence genes using the eazyplex® hv- *K. pneumoniae* assay (Amplex Diagnostics GmbH, Gars-Bahnhof, Germany). This loop-mediated isothermal amplification (LAMP) assay enabled screening for presence of the marker genes aerobactin, salmochelin, colibactin, yersiniabactin, *rmpA* and *rmpA2*. This strategy was chosen based on the convincing results we obtained in a pilot study to establish a practicable surveillance approach for hypervirulence traits in microbiological routine diagnostics.

**Fig. 1:**
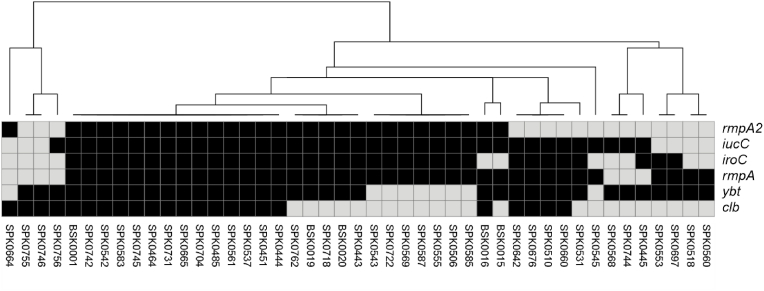
Visual presentation of hypervirulence-associated marker genes detected in the *K. pneumoniae* isolates of the pilot cross-border surveillance study. Absent genes were depicted as grey. Visualization was realized with the Morpheus online tool (https://software.broadinstitute.org/morpheus), using hierarchical clustering approach with Jaccard distance and UPGMA approach.

As suggested by Han et al., solely isolates with n ≥ 2 virulence genes were recognized as putative hvKp (=genetically hvKp) in the following. From 4398 string-tested isolates on both study sites, 45 isolates were identified as genetically hvKp. The virulence gene content varied for the isolates (Fig. 1).

As shown in Fig. 1, most common genes were aerobactin (*iucC*; n = 38) and *rmpA* (n = 38) genes, followed by yersiniabactin (*ybt*; n = 36) and salmochelin (*iroC*; n = 34). Colibactin (*clb*; n = 23) and *rmpA* (n = 29) genes were less frequently detected. Clustering revealed different combinations of marker genes and showed that most isolates carried n ≥ 4 genes. The isolates from centre 2 (BSK) carried all n ≥ 4 genes. Two Austrian and one German isolate were ESBL-producers, the other 42 putative hvKp were susceptible to antibiotics.

The testing revealed the different prevalence of putative hvKp isolates in the two study centres, as shown in Table 1.

**Table 1:** Results of the cross-border hvKp-testing in two tertiary hospitals in Germany and Austria in 2023.

Study centre	Country	Hospitalized patients	String-tested isolates	String-test positive isolates^a^	Percentage putative hvKp [number]	Estimated prevalence^b^	Nosocomial acquisition of putative hvKp	Hospitalization of patients with putative hvKp	Proportion female patients with putative hvKp	Age of patients with putative hvKp [range]
Center 1	Germany	81,665	3115	269	14.9 % [40/269]	1.28 %	42.5 % [17/40]	Mean: 8.6 d	30.0 % [12/40]	Median: 64.5 y [35–95]
								Median: 2 d		
Center 2	Austria	69,979	1283	22	22.7 % [5/22]	0.39 %	20 % [1/5]	Mean: 33.3 d	60.0 % [3/5]	Median: 72 y [0–74]
								Median: 11 d		

a Excluding multiple *K. pneumoniae* isolated the same patient at the same time.

b Regarding all string-tested clinical isolates of *K. pneumoniae* per study centre.

In the German centre, 8-times more isolates with n ≥ 2 virulence genes were identified, while only 2.4-times more isolates were initially tested. This results also in a 3.3-times higher prevalence estimation in the German hospital, compared to the Austrian. Interestingly, 33 of 45 putative hvKp (73.3 %) isolates originated from male patients, whereas the proportion of female patients differed for the two centres. The median patient age were comparable. Interestingly, the hospitalization of patients with putative hvKp differed between the centres, with longer hospitalization days in centre 2. In contrast, the rate of nosocomial acquisition of putative hvKp was twice as high in centre 1 as in centre 2. Further, isolates from centre 1 included four cases of liver abscesses, resulting in a prevalence of 0.13 % regarding all *K. pneumoniae*, or 1.49 % of string-test positive isolates in 2023. Overall, 10 % of putative hvKp identified by string-test and LAMP, could be defined as clinical hvKp cases.

Here, we showed that by using a testing approach for identification of putative hvKp is possible in clinical routine diagnostics. In addition, the testing strategy enabled the epidemiological assessment of hvKp liver abscess cases in the context of all isolates and the knowledge about hypervirulence gene carriage in the *K. pneumoniae* population. The approach using an initial string-test is a limitation, but it selected for capsule-regulator marker genes that were identified as important marker of hvKp isolates in Germany [3]. Most positively tested by LAMP, also carried the *rmpA* and/or *rmpA2* gene. Unfortunately, no sequencing-data could be included at this time, so no information about sequence type and serotype are available.

Even regarding identical study design and inclusion criteria in both study centres, the epidemiological situation of hvKp might vary a lot comparing different countries as it is known for multi-drug resistant pathogens [4]. Our approach for microbiological routine testing poses a diagnostics-focused surveillance approach and useful addition to investigations based on patient case presentation. Further, multicentre studies are warranted to give more insights in the epidemiology of hvKp.

## CRediT authorship contribution statement

**Bernd Neumann:** Writing – review & editing, Writing – original draft, Visualization, Validation, Methodology, Investigation, Funding acquisition, Formal analysis, Data curation, Conceptualization. **Sophie Wilhelm:** Writing – review & editing, Formal analysis. **Jan Marco Kern:** Writing – review & editing, Validation, Funding acquisition, Data curation. **Joerg Steinmann:** Writing – review & editing, Validation, Project administration, Funding acquisition.

## Ethics approval

Not required.

## Sequence information

Not applicable.

## Funding

This project was financially supported by an intramural cross-location research funding “Bridging Nuremberg-Salzburg” of the Paracelsus Medical University; as part of the project “Surveillance and molecular investigations of hypervirulent clinical isolates of *Klebsiella pneumoniae*” (PMU-RIF no. FMS_W_088.22-H).

## Declaration of competing interest

The authors declare that they have no known competing financial interests or personal relationships that could have appeared to influence the work reported in this paper.
